# Cost-effectiveness of six strategies for *Helicobacter pylori *diagnosis and management in uninvestigated dyspepsia assuming a high resource intensity practice pattern

**DOI:** 10.1186/1472-6963-10-344

**Published:** 2010-12-21

**Authors:** Kyland P Holmes, John C Fang, Brian R Jackson

**Affiliations:** 1 ARUP Laboratories, Salt Lake City, Utah, USA; 2 Department of Medicine, University of Utah, Salt Lake City, Utah, USA; 3 Department of Pathology, University of Utah, Salt Lake City, Utah, USA, ARUP Laboratories, Salt Lake City, Utah, USA

## Abstract

**Background:**

Initial assessment of dyspepsia often includes noninvasive testing for *Helicobacter pylori *infection. Commercially available tests vary widely in cost and accuracy. Although there is extensive literature on the cost-effectiveness of *H. pylori *treatment, there is little information comparing the cost-effectiveness of various currently used, noninvasive testing strategies.

**Methods:**

A Markov simulation was used to calculate cost per symptom-free year and cost per correct diagnosis. Uncertainty in outcomes was estimated using probabilistic sensitivity analysis.

**Results:**

Under the baseline assumptions, cost per symptom-free year was $122 for empiric proton pump inhibitor (PPI) trial, and costs for the noninvasive test strategies ranged from $123 (stool antigen) to $129 (IgG/IgA combined serology). Confidence intervals had significant overlap.

**Conclusions:**

Under our assumptions for how testing for *H. pylori *infection is employed in United States medical practice, the available noninvasive tests all have similar cost-effectiveness between one another as well as with empiric PPI trial.

## Background

Many diagnostic scenarios require a physician to choose from among a set of related diagnostic tests. For *Helicobacter pylori *infection in the setting of dyspepsia, diagnostic options include serologic tests, a stool antigen test, a urea breath test, and invasive methods, such as endoscopy with biopsy. These options vary with regard to cost, convenience, and accuracy. The decision about which test to order may ultimately be a significant driver of downstream economic costs and quality-of-care outcomes. The American Gastroenterological Association (AGA) in 2005 recommended that to diagnose *Helicobacter pylori *infection in patients with dyspepsia, physicians should use either the stool antigen test or the urea breath test.[1,2] Serologic tests were specifically not recommended due to inferior sensitivity and specificity. The American College of Gastroenterology (ACG) issued a similar guideline in 2007 that discussed the tradeoffs among available tests but left the test choice up to physician judgment.[3] The testing volume for *H. pylori *at a national reference laboratory (ARUP Laboratories, Salt Lake City, Utah) suggests that serology is more widely used than would be expected under the AGA-recommended approach. ARUP received approximately four times as many orders for *H. pylori *serology between April 1, 2007, and March 31, 2008, than for either stool or breath tests. For this reason, serology is included in the current analysis.

A number of published articles address the cost-effectiveness of *H. pylori *management, but most of these primarily compare treatment options after a patient has already been diagnosed with *H. pylori*.[4-25] Others compare early endoscopy to empiric therapy and/or noninvasive testing.[26-32] Still others compare the effectiveness within a small subset of tests of diagnosing *H. pylori *infection or of determining the efficacy of treatment.[27-30,33-43] At least one article has assessed cost-effectiveness of multiple noninvasive tests before and after diagnosis.[44] Given that "test and treat" using noninvasive tests is a common approach to uninvestigated dyspepsia in the United States, the specific noninvasive test strategy becomes a key decision point. We therefore compared the cost-effectiveness of some of the most common noninvasive testing strategies for *H. pylori *along with empiric proton pump inhibitor (PPI) trial in the management of uninvestigated dyspepsia.

## Methods

**Figure 1:**
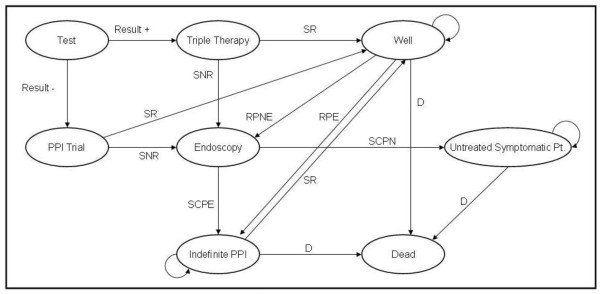
**Diagram of *H. pylori *Markov model**. SR = Symptoms resolve. SNR = Symptoms do not resolve. RPE = Relapse, PPI initially effective. RPNE = Relapse, PPI not initially effective. D = Death from all causes. SCPE = Symptoms continue, PPI initially effective. SCPN = Symptoms continue, PPI not initially effective.

This study compared six diagnostic strategies [Table 1] for initial management of patients with dyspepsia. The first step of strategies 1 through 5 was a different noninvasive test, and empiric PPI therapy was included for completeness as a sixth strategy. In order to calculate the impact (both cost and benefit) of the choice of diagnostic test we had to first create a model of the expected care process for these patients. We intentionally did not use an idealized care process, but rather modeled it to reflect typical local practice for managing dyspepsia (Figure 1). We also believe it to be a reasonable representation of a practice patterns for dyspepsia across much of the US.

**Table 1:** Description of strategies modeled.

Strategy	Description
IgG/IgA	Begin with *H. pylori *IgG and IgA tests. If either is positive, do triple therapy. If both are negative, do PPI trial.
IgG	Begin with *H. pylori *IgG test. If positive, do triple therapy. If negative, do PPI trial.
Stool Antigen	Begin with *H. pylori *stool antigen test. If positive, do triple therapy. If negative, do PPI trial.
IgG with reflex to stool Antigen	Begin with IgG test. If positive, confirm with stool antigen detection. If both tests are positive, do triple therapy. Otherwise, do PPI trial.
Breath Test	Begin with *H. pylori *urea breath test. If positive, do triple therapy. If negative, do PPI trial.
PPI trial	Skip noninvasive testing and begin instead with PPI trial.

The primary measure of outcome was the cost (US$) per symptom-free year. As the length of triple therapy or PPI trial is typically 14 days and this is the event of shortest duration in the model other than initial testing, the Markov cycle was defined as two weeks.

Key assumptions in this model include:

1. All patients enter the model with uninvestigated dyspepsia. There is some probability that the patient has an *H. pylori *infection, peptic ulcer(s), or both.

2. In keeping with AGA and ACG guidelines, the model considers only patients younger than 55 years of age who lack alarm features, such as bleeding or weight loss. Accordingly, the model does not consider the downstream costs or the medical consequences of gastric cancer, perforated ulcers, and other potentially life-threatening conditions.

3. Any patient who tests positive for *H. pylori *will be administered a course of standard triple therapy (clarithromycin, amoxicillin, and lansoprazole).

4. If there is no relief of symptoms after initial management, or if symptoms recur, he or she will go on to receive an endoscopy with a biopsy. This assumption was followed not in an attempt to reflect evidence-based practice, but rather in an attempt to reflect a "typical" approach to dyspepsia management.

5. Following endoscopy, if triple therapy or empiric PPI had been initially effective in relieving symptoms of dyspepsia, and if symptoms remained unresolved or recurred, the patient will receive long-term PPI therapy. As above, this assumption was followed in an attempt to reflect typical practice patterns, rather than evidence-based practice per se.

6. Patients remain in the model for the duration of their life.

The scenarios above were modeled by a Markov chain Monte Carlo (MCMC) method of statistical analysis using the TreeAge Pro software package (TreeAge Software, Inc., Williamstown, Massachusetts). A simplified graphical representation of the model is given in Figure 1. Variable inputs for the model are listed in Table 2. The outcomes measured were total cost of diagnosis and treatment in U.S. dollars and the amount of time spent symptom-free. The cost perspective taken was societal.

**Table 2:** Variables employed in the model.

Variable	Mean	Distribution	95th percentile *	Reference(s)
*H. pylori *prevalence	0.23	Uniform	0.05 - 0.4	[3,44,46]
Initial ulcer status	0.10	Beta	0.025-.022	[1,2]
Cost of IgA serology	$29	Gamma	12.53-51.34	**
Cost of IgG serology	$29	Gamma	12.53-51.34	**
Cost of stool Ag detection	$21	Gamma	6.20-44.56	**
Cost of urease breath test	$133	Gamma	28.81-316.05	**
Cost of endoscopy w/biopsy and pathologist time	$511	Gamma	156.21-2552.46	***
Cost of eradication therapy	$355	Gamma	186.55-576.02	***
Cost of 2-week PPI therapy	$40	Gamma	10.92-87.51	***
Breath test sensitivity	0.95	Triangular	0.90 - 0.98	[28,34,39,41,44]
Breath test specificity	0.96	Triangular	0.94 - 0.99	[28,34,39,41,44]
IgA serology sensitivity	0.85	Triangular	0.79 - 0.90	[27,29-31,38,41,44,47-51]
IgA serology specificity	0.79	Triangular	0.65 - 0.85	[27,29-31,38,41,44,47-51]
IgG serology sensitivity	0.85	Triangular	0.79 - 0.90	[27,29-31,38,41,44,47-51]
IgG serology specificity	0.79	Triangular	0.65 - 0.85	[27,29-31,38,41,44,47-51]
Stool Ag sensitivity	0.93	Triangular	0.90 - 0.99	[27,30,33-35,37,40-44,52-56]
Stool Ag specificity	0.92	Triangular	0.90 - 0.99	[27,30,33-35,37,40-44,52-56]
Probability of relief of symptoms after two weeks of triple therapy (NUD§)	0.53	Beta	0.28-0.66	[57]
Probability of relief of symptoms after two weeks of triple therapy (PUD§§)	0.49	Beta	0.28-0.70	[11]
Probability of relief of symptoms after two-week PPI trial (NUD)	0.40	Beta	0.40-0.78	Expert opinion§§§
Probability of relief of symptoms after two-week PPI trial (PUD)	0.32	Beta	0.47-0.85	[11]
Probability of relapse at one year after eradication therapy (NUD)	0.32	N/A	N/A	[57]
Probability of relapse at one year after eradication therapy (PUD)	0.37	N/A	N/A	[11]

*Calculated by taking 100,000 samples from each distribution.

**These estimates are based on 2009 Medicare reimbursement rates, where the mean is the 2009 midpoint national reimbursement rate as published in the 2009 Clinical Diagnostic Laboratory Fee Schedule, which is available at http://www.cms.hhs.gov/ClinicalLabFeeSched/.

***These figures are based on Medicare reimbursement rates, which are current for 2009 CPT codes and are available from the American Medical Association.

§Non-ulcer dyspepsia.

§§Peptic ulcer disease.

§§§John C. Fang, MD, Associate Professor of Gastroenterology, University of Utah, Salt Lake City, Utah

Probabilities employed in the model were based on published literature where available. Expert opinion was provided by one of the coauthors (JCF). Baseline costs of tests and treatments were based on 2009 national midpoint Medicare reimbursement rates. Second-order probabilistic sensitivity analysis, simulating 250 trials involving 10,000 patients each, was undertaken to measure the extent to which parameter uncertainty might affect the model outcomes. Incremental cost-effectiveness ratios (ICERs) were calculated based on a single simulated cohort of 500,000 patients using empiric PPI trial (i.e., no testing) as the baseline for comparison.

## Results

The cost-effectiveness ratios for the six initial management strategies were similar (Table 3); stool antigen and empiric PPI trial were essentially equivalent, and both strategies were mildly superior to the remaining diagnostic test strategies. Some previously published cost-effectiveness analyses have reported results in the form of mean cost per correct diagnosis; to allow easier comparison with these we provide our results according to this measure in Table 4. None of these results were sensitive to changes in prevalence of *H. pylori *(5% to 40%).

**Table 3:** Cost-Effectiveness ratios for each strategy modeled.

Strategy	Cost per symptom free year (95% CI*)
Empiric PPI Trial	122.13 (120.00-124.88)
Stool Ag	123.23 (120.68-125.58)
IgG serology	125.76 (123.18-128.27)
IgG serology w/reflex to Stool Ag	126.17 (123.43-128.08)
Breath test	128.31 (125.69-130.72)
IgG/IgA binary serology	129.04 (126.43-131.48)

Numerical values are in $US per symptom-free year.

**Table 4:** Cost per correct diagnosis for each strategy modeled

Testing Strategy	Average Cost per Correct Diagnosis
Stool Ag	$2767.85
Breath test	$2825.24
IgG Serology	$3371.91
IgG serology w/reflex to Stool Ag	$3373.39
IgG/IgA binary serology	$4061.91

## Discussion

Assuming that this model and its assumptions reasonably reflect U.S. clinical practice, it appears that the initial choice of noninvasive testing strategy does not have a significant influence on the overall cost-effectiveness of care for patients presenting with previously uninvestigated dyspepsia. This finding holds even when the prevalence of *H. pylori *infection is varied over a wide range (5% to 40%).

These findings may seem surprising; it seems intuitive that more accurate testing should lead to improved outcomes and lower overall health spending, provided that the cost of the test itself is reasonable. The key, though, is the set of baseline assumptions about how the tests are used in U.S. clinical practice. In particular, we assumed that in the absence of symptomatic relief, physicians would move fairly quickly to definitive diagnosis (endoscopy with biopsy), thus reducing or even negating the impact of the original noninvasive test (or lack thereof). To use a more extreme illustration, a consequence of our underlying model is that the diagnostic test could be replaced with a random number generator without significantly impacting cost-effectiveness. In settings where endoscopy is less widely employed, the decision about which noninvasive test to order for a patient would likely have a larger economic and clinical impact, depending on the test chosen.

Vakil et al. estimated the cost-effectiveness of a similar set of noninvasive testing strategies in a 2000 paper [44]. In their model, cost per correct diagnosis using the antibody and stool tests ranged from $90 to $127. The antibody tests incurred the lowest cost per correct diagnosis at all levels of prevalence that they modeled (30%, 60%, and 90%). The stool test was more expensive at low and intermediate prevalence, but with much higher diagnostic accuracy (~93%). A key distinction of our model is that we used cost per symptom-free year rather than cost per correct diagnosis as our primary measure, although we did include the latter in our results for comparison. This distinction is significant. First, the clinical goal is typically relief of symptoms rather than diagnosis per se. A patient is unlikely to undergo follow-up when symptoms have been alleviated, as in the empiric PPI trial or a PPI trial after eradication treatment. The impact of the diagnosis can only be truly assessed in the context of the resulting clinical actions. Therefore, the cost *at *diagnosis does not reflect the total cost of care. For example, a patient can have an incorrect noninvasive test result, but if the patient still achieves long-term relief of symptoms without incurring too much expense, then from that patient's

perspective, no great harm has been done. We thus believe that cost per symptom-free year is a more appropriate cost-effectiveness measure than cost per correct diagnosis. In a larger sense, our study illustrates some of the challenges in assessing cost-effectiveness of diagnostic tests, as well as the importance of doing so. Because testing is an upstream process, the clinical and economic impacts of diagnostic tests are expressed primarily in the downstream clinical actions. The result from a $10 test, for example, might in some cases make the difference between whether to provide a $10,000 course of chemotherapy or a surgical procedure. Or, in a different scenario, that same $10 test might simply be a waste of $10, if the same clinical actions took place regardless of the test result. Superior sensitivity and/or specificity does not guarantee better patient outcomes once the test is placed in the larger context of patient management. In other words, cost-effectiveness is less an inherent property of a particular test than it is a property of the decision-making algorithm in which that test is employed.[45]

## Limitations

The primary limitation of our analysis was our set of clinical assumptions. These results should not be considered to have validity outside of those assumptions. This includes both the assumed practice pattern (Figure 1) and the numbers (costs and probabilities). In clinical settings that do not fit these assumptions our results may not apply. Another limitation is that we did not present our results in terms of quality-adjusted life years (QALYs), partly because we did not find any widely used figures for quality-of-life measurements for patients with dyspepsia, and partly because the existing literature on cost-effectiveness of *H. pylori *diagnostic tests does not appear to rely heavily on QALY analysis. Also, as stated above, our model was based entirely on dyspepsia relief and did not consider more serious illnesses such as a perforated ulcer, gastric cancer, etc.

Finally, our analysis relied heavily on the findings of a Cochrane systematic review[18] in which *H. pylori *eradication was found to have only a very small clinical benefit for the average patient with nonulcer dyspepsia. Our findings may thus not apply to patient subsets for which eradication therapy could be shown to have a larger average benefit.

## Conclusions

In this model of *H. pylori *diagnosis and treatment, the choice of initial noninvasive test did not have a significant impact on cost or quality outcome. This is likely attributable to the assumption of a high resource intensity practice environment. In practice settings where endoscopy is less available and/or less readily employed, these findings may not apply.

## Authors' contributions

KPH constructed and debugged the cost-effectiveness model, performed the literature review, and drafted the initial manuscript with figures and tables. JCF provided clinical expertise and assisted in interpreting the findings. BRJ conceived the study and provided oversight and editorial review. All authors have read and approved the final version of the manuscript.

Financial support: Salary support for Kyland Holmes and Brian Jackson provided by ARUP Laboratories, a nonprofit enterprise of the University of Utah

Potential competing interests: None.

## Pre-publication history

The pre-publication history for this paper can be accessed here:

http://www.biomedcentral.com/1472-6963/10/344/prepub
